# The crystal structures of iron and cobalt pyridine (py)–sulfates, [Fe(SO_4_)(py)_4_]_*n*_ and [Co_3_(SO_4_)_3_(py)_11_]_*n*_

**DOI:** 10.1107/S2056989018007557

**Published:** 2018-05-31

**Authors:** Duyen N. K. Pham, Mrittika Roy, Ava Kreider-Mueller, James A. Golen, David R. Manke

**Affiliations:** a Department of Chemistry and Biochemistry, University of Massachusetts Dartmouth, 285 Old Westport Road, North Dartmouth, MA 02747, USA

**Keywords:** crystal structure, pyridine, sulfate, transition metals, crystal field theory, coordination chemistry

## Abstract

The solid-state structures of two metal–pyridine–sulfate compounds, namely *catena*-poly[[tetra­kis­(pyridine-κ*N*)iron(II)]-μ-sulfato-κ^2^ *O*:*O*′], [Fe(SO_4_)(C_5_H_5_N)_4_]_*n*_, (**1**), and *catena*-poly[[tetra­kis­(pyridine-κ*N*)cobalt(II)]-μ-sulfato-κ^2^ *O*:*O*′-[tetra­kis­(pyridine-κ*N*)cobalt(II)]-μ-sulfato-κ^3^ *O*,*O*′:*O*′′-[tris­(pyridine-κ*N*)cobalt(II)]-μ-sulfato-κ^2^ *O*:*O*′], [Co_3_(SO_4_)_3_(C_5_H_5_N)_11_]_*n*_, (**2**), are reported. The iron compound (**1**) displays a polymeric structure, with infinite chains of Fe^II^ atoms adopting octa­hedral N_4_O_2_ coordination environments that involve four pyridine ligands and two bridging sulfate ligands. The cobalt compound (**2**) displays a polymeric structure, with infinite chains of Co^II^ atoms. Two of the three Co centers have an octa­hedral N_4_O_2_ coordination environment that involves four pyridine ligands and two bridging sulfate ligands. The third Co center has an octa­hedral N_3_O_3_ coordination environment that involves three pyridine ligands, and two bridging sulfate ligands with one sulfate chelating the cobalt atom.

## Abstract 2

The crystal structures of two first-row transition metal (Fe and Co) pyridine–sulfate complexes are presented. The compounds demonstrate infinite chains of metal pyridine units connected by bridging sulfate anions.

## Chemical context   

The first reports of a pyridine–sulfato–metal complex were in the late 19th century (Reitzenstein, 1894 ▸; Reitzenstein, 1898 ▸), and this work played a significant role in the Werner–Jørgensen controversy (Howe, 1898 ▸). While most early work in coordination chemistry was based upon ammonia complexes, the demonstration of the existence of similar complexes with other organic bases such as pyridine was an important contribution to the field. Despite the long history of these complexes, and their contributing role in the development of coordination chemistry, their crystallographic characterization is limited.
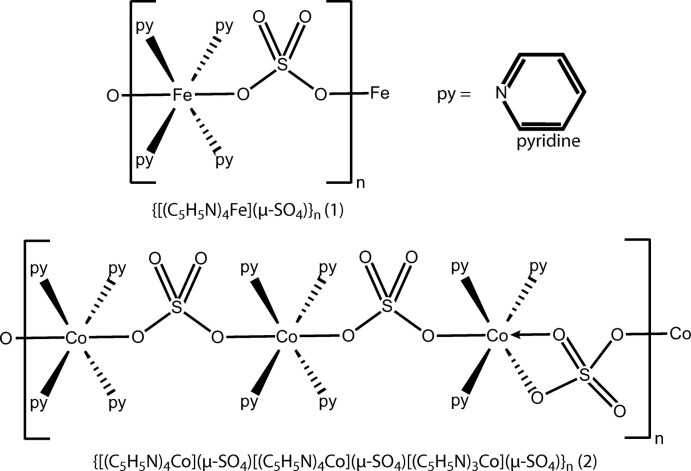


Against this backdrop, our lab has recently begun to study the solid-state structures of transition-metal pyridine complexes. We have recently reported the structures of nickel, copper and zinc pyridine sulfates, which showed varying coordination geometries consistent with those predicted by crystal field theory (Roy *et al.*, 2018 ▸). Herein, we expand this series by presenting the crystal structures of the iron–pyridine–sulfate (**1**) and the cobalt–pyridine–sulfate (**2**) complexes.

## Structural commentary   

**Figure 1:**
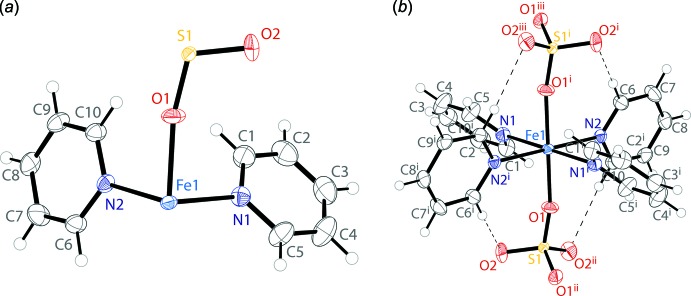
The mol­ecular structure of compound (**1**), including (*a*) the asymmetric unit and (*b*) the coordination environment of Fe1. Displacement ellipsoids are drawn at the 50% probability level. H atoms are drawn as spheres of arbitrary radius. C—H⋯O inter­actions (Table 1 ▸) are shown as dashed lines. [Symmetry codes: (i) −*x*, −*y*, −*z* (ii) −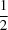 − *x*, *y*, −*z* (iii) 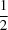 + *x*, −*y*, *z*]

In the yellow crystals of (**1**), the asymmetric unit consists of two pyridine mol­ecules and one half of a sulfate anion coordinated to an iron atom sitting on an inversion center (Fig. 1 ▸ *a*). When grown out, the iron displays an octa­hedral coordination environment (Fig. 1 ▸ *b*). There is a square-planar tetra­pyridine iron unit, with FeN_4_ planarity enforced by the inversion. The octa­hedral coordination is completed by two sulfate ions that bind *trans* to each other. The *cis* N—Fe—N angles have values of 86.44 (4) and 93.56 (4)° and the *cis* O—Fe—N angles have values ranging from 88.12 (4) to 91.88 (4)°. The pyridine rings are rotated from the FeN_4_ plane by dihedral angles of 44.03 (1) and 78.20 (1)°. The 78.20 (1)° angle is constrained by two C—H⋯O inter­actions with the *trans* sulfates (Table 1 ▸).

**Figure 2:**
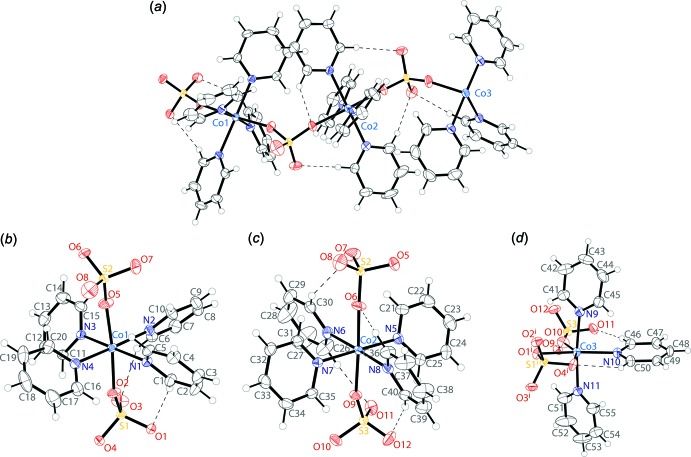
The mol­ecular structure of compound (**2**), including (*a*) the asymmetric unit, (*b*) the coordination environment of Co1, (*c*) the coordination environment of Co2 and (*d*) the coordination environment of Co3. Displacement ellipsoids are drawn at the 50% probability level. H atoms are drawn as spheres of arbitrary radius. C—H⋯O inter­actions (Table 2 ▸) are shown as dashed lines. [Symmetry codes: (i) 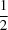 − *x*, 1 − *y*, −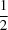 + *z*]

In the pink crystals of (**2**), the asymmetric unit consists of three cobalt atoms, eleven coordinated pyridine mol­ecules, and three sulfate anions (Fig. 2 ▸ *a*). There are three crystallographically independent cobalt atoms, with Co1 (Fig. 2 ▸ *b*) and Co2 (Fig. 2 ▸ *c*) displaying octa­hedral N_4_O_2_ coordination environments, and Co3 showing an octa­hedral N_3_O_3_ coordination environment (Fig. 2 ▸ *d*).

Co1 is part of a tetra­pyridine cobalt unit, with the CoN_4_ plane showing a maximum deviation from planarity of 0.047 Å. The octa­hedral coordination is completed by two sulfate anions that bind *trans* to each other. The *cis* N—Co—N angles have values ranging from 87.06 (10) to 93.21 (9)°, and the O—Co—O angle is 174.62 (9)°. The four pyridine rings are rotated from the CoN_4_ plane by dihedral angles of 37.51 (1), 45.21 (1), 56.40 (1) and 56.92 (1)°. Two of the rings form one C—H⋯O inter­action each with the sulfate oxygen atoms (Table 2 ▸).

Co2 is also part of a tetra­pyridine cobalt unit, with the CoN_4_ plane showing a maximum deviation from plarity of 0.007 Å. The octa­hedral coordination is completed by two sulfate anions that bind *trans* to each other. The *cis* N—Co—N angles have values ranging from 85.15 (9) to 93.19 (9)°, and the O—Co—O angle is 175.16 (9)°. The four pyridine rings are rotated from the CoN_4_ plane by dihedral angles of 55.37 (1), 65.88 (1), 67.08 (1) and 68.07 (1)°. Two of the rings are involved in two C—H⋯O inter­actions each with the sulfate oxygen atoms (Table 2 ▸).

Unlike the other two metal centers, Co3 has an N_3_O_3_ coordination environment, possessing a meridional arrangement. It is part of a tri­pyridine cobalt unit, with a CoN_3_ plane showing a maximum deviation from planarity of 0.021 Å. The octa­hedral coordination is completed by two bridging sulfate anions (one of which chelating through the oxygen atoms O1 and O4) that form a CoO_3_ plane with a maximum deviation from planarity of 0.029 Å. The meridional CoN_3_ and CoO_3_ planes are rotated relative to one another by an angle of 88.93 (1)°. The *cis* N—Co—N angles have values of 86.76 (10) and 87.52 (9)°. The chelating sulfate exhibits an O—Co—O bite angle of 65.36 (7)° and another *cis* O—Co—O angle of 88.63 (8)°. The three pyridine rings are rotated from the CoN_3_ plane by dihedral angles of 31.855 (2), 44.111 (3) and 82.863 (4)°. The 82.863 (4)° angle is constrained by two C—H⋯O inter­actions with sulfate oxygen atoms (Table 2 ▸).

## Supra­molecular features   

**Figure 3:**
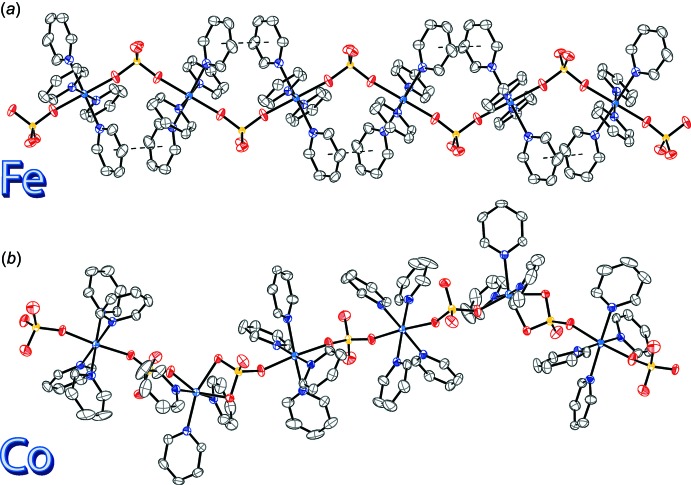
The infinite chains of (*a*) compound (**1**) along [100] and (*b*) compound (**2**) along [001]. Displacement ellipsoids are drawn at the 50% probability level. H atoms are omitted for clarity. π–π inter­actions are shown as dashed lines.

In compound (**1**), the Fe^II^ atoms are linked together into infinite chains along the [100] direction through the sulfate ligands *via* O—S—O bridges (Fig. 3 ▸ *a*). Between each successive tetra­pyridine iron unit are found parallel slipped π–π inter­actions [inter-centroid distance: 3.651 (1) Å, inter-planar distance: 3.607 (1) Å, slippage: 0.570 (1) Å].

**Figure 4:**
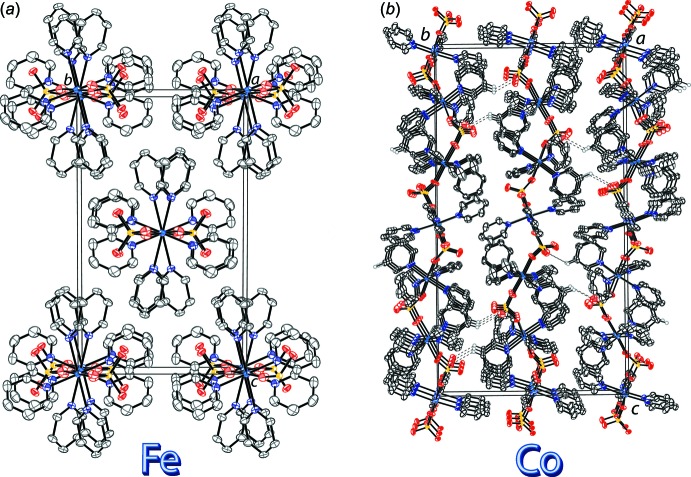
The packing of (*a*) compound (**1**) and (*b*) compound (**2**) along the *a* axis. Displacement ellipsoids are drawn at the 50% probability level. In (**2**), H atoms are omitted for clarity in compound (*1*). H atoms involved in hydrogen bonding between chains are drawn as spheres of arbitrary radius, with the other H atoms omitted for clarity. C—H⋯O inter­actions (Table 2 ▸) are shown as dashed lines.

In compound (**2**), the Co^II^ atoms linked together into infinite chains along the [001] direction through the sulfate ligands (Fig. 3 ▸ *b*). No π–π inter­actions are observed in this crystal. There are two C—H⋯O inter­actions between chains [C4—H4⋯O11, *d*(C⋯O) = 3.158 (4) Å and C24—H24⋯O7, *d*(C⋯O) = 3.322 (4) Å] that connect the chains in three dimensions (Table 2 ▸). The packing of both compounds is shown in Fig. 4 ▸.

## Database survey   

Though complexes of this form have been known for more than a century, their crystallographic characterization has been limited. Prior to our report earlier this year, there were only two structures in the literature of metal–pyridine–sulfates with no other ligands or components (Cotton & Reid, 1984 ▸; Memon *et al.*, 2006 ▸). There are a number of closely related structures that have been reported, particularly transition-metal–aqua–pyridine–sulfate complexes. Six of these are found in the literature (Ali *et al.*, 2005 ▸; Castiñeiras & García-Santos, 2008 ▸; Cotton *et al.*, 1994 ▸; Kožíšek *et al.*, 1989 ▸; Shi *et al.*, 2009 ▸; Zhang, 2004 ▸). The metrical parameters in the reported structures are consistent with those seen in the metal–pyridine–triflates (Haynes *et al.*, 1986 ▸).

In a report earlier this year, we presented the structures of the metal–pyridine–sulfates of nickel, copper and zinc. It was of note that these three structures exhibited different coordination geometries, consistent with the crystal field stabilization energies (CFSE) associated with their *d*-electron count: *d* ^8^ nickel is octa­hedral, *d* ^9^ copper is square pyramidal, and *d* ^10^ zinc is both tetra­hedral and octa­hedral. The structures reported here both exhibit octa­hedral coordination environments. For *d* ^6^ iron, the observed octa­hedral environment gives a CFSE of 4 Dq, while the preferred geometry might be square pyramidal with a CFSE of 4.67 Dq. Similarly for *d* ^7^ cobalt, the observed octa­hedral environment gives a CFSE of 8 Dq, while the preferred geometry might once again be square pyramidal with a CFSE of 9.14 Dq. The difference between octa­hedral and square pyramidal in these two compounds is small compared to the 3.14 Dq difference for *d* ^9^ copper, where a square-pyramidal geometry is observed. With such small electronic preferences, the impact of weaker inter­actions (π–π and C—H⋯O) and steric effects could play significant roles in determining the observed coordination environments.

## Synthesis and crystallization   

Approximately 25 mg of each metal sulfate [iron sulfate hepta­hydrate (J. T. Baker), cobalt sulfate hepta­hydrate (J. T. Baker)] were dissolved in pyridine (3 mL, Fisher Chemical) in a 20 mL vial under an atmosphere of di­nitro­gen. In the cobalt case, 0.1 mL of water was also added. The vials were heated to 353 K for 24–48 h, after which single crystals suitable for X-ray diffraction studies were isolated.

## Refinement   

Crystal data, data collection and structure refinement details are summarized in Table 3 ▸. All structure solutions were obtained by intrinsic phasing. All non-hydrogen atoms were refined anisotropically (*SHELXL*) by full-matrix least squares on *F* ^2^. Hydrogen atoms were placed in calculated positions and then refined with a riding model with C—H bond lengths of 0.95 Å and with isotropic displacement parameters set to 1.20 *U*_eq_ of the parent C atom.

## Supplementary Material

Crystal structure: contains datablock(s) 1, 2. DOI: 10.1107/S2056989018007557/sj5556sup1.cif

Structure factors: contains datablock(s) 1. DOI: 10.1107/S2056989018007557/sj55561sup4.hkl

Structure factors: contains datablock(s) 2. DOI: 10.1107/S2056989018007557/sj55562sup5.hkl

CCDC references: 1844143, 1844142

Additional supporting information:  crystallographic information; 3D view; checkCIF report

## Figures and Tables

**Table 1:** Hydrogen-bond geometry (Å, °) for (**1**)

*D*—H⋯*A*	*D*—H	H⋯*A*	*D*⋯*A*	*D*—H⋯*A*
C6—H6⋯O2^i^	0.95	2.49	3.4296 (19)	169
C10—H10⋯O2^ii^	0.95	2.42	3.3621 (19)	171

Symmetry codes: (i) 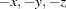; (ii) 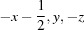.

**Table 2:** Hydrogen-bond geometry (Å, °) for (**2**)

*D*—H⋯*A*	*D*—H	H⋯*A*	*D*⋯*A*	*D*—H⋯*A*
C1—H1⋯O1	0.95	2.56	3.421 (4)	150
C1—H1⋯O2	0.95	2.58	3.066 (4)	112
C4—H4⋯O11^i^	0.95	2.47	3.158 (4)	129
C6—H6⋯O3	0.95	2.48	3.263 (4)	140
C15—H15⋯O5	0.95	2.47	2.967 (4)	113
C24—H24⋯O7^ii^	0.95	2.59	3.322 (4)	134
C26—H26⋯O11	0.95	2.40	3.343 (4)	171
C30—H30⋯O6	0.95	2.51	3.079 (4)	119
C30—H30⋯O7	0.95	2.50	3.161 (4)	126
C31—H31⋯O6	0.95	2.59	3.107 (4)	115
C35—H35⋯O9	0.95	2.36	2.936 (4)	119
C36—H36⋯O6	0.95	2.41	3.003 (4)	121
C40—H40⋯O12	0.95	2.43	3.352 (4)	163
C46—H46⋯O11	0.95	2.30	3.225 (4)	166
C50—H50⋯O4^iii^	0.95	2.49	3.132 (4)	125
C51—H51⋯O10	0.95	2.46	3.019 (4)	117

Symmetry codes: (i) 
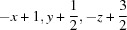
; (ii) 
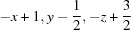
; (iii) 
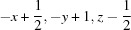
.

**Table 3:** Experimental details

	(**1**)	(**2**)
Crystal data		
Chemical formula	[Fe(SO_4_)(C_5_H_5_N)_4_]	[Co_3_(SO_4_)_3_(C_5_H_5_N)_11_]
*M*_r_	468.31	1335.07
Crystal system, space group	Monoclinic, *I*2/*a*	Orthorhombic, *P*2_1_2_1_2_1_
Temperature (K)	200	200
*a*, *b*, *c* (Å)	11.8259 (10), 10.0847 (9), 17.264 (2)	9.4583 (5), 18.0344 (12), 33.088 (2)
α, β, γ (°)	90, 102.569 (2), 90	90, 90, 90
*V* (Å^3^)	2009.6 (3)	5644.0 (6)
*Z*	4	4
Radiation type	Mo *K*α	Mo *K*α
μ (mm^−1^)	0.89	1.06
Crystal size (mm)	0.28 × 0.20 × 0.20	0.24 × 0.22 × 0.20

Data collection		
Diffractometer	Bruker D8 Venture CMOS	Bruker D8 Venture CMOS
Absorption correction	Multi-scan (*SADABS*; Bruker, 2016 ▸)	Multi-scan (*SADABS*; Bruker, 2016 ▸)
*T*_min_, *T*_max_	0.397, 0.429	0.394, 0.429
No. of measured, independent and observed [*I* > 2σ(*I*)] reflections	25476, 1917, 1760	80759, 10744, 9925
*R*_int_	0.029	0.037
(sin θ/λ)_max_ (Å^−1^)	0.612	0.612

Refinement		
*R*[*F* ^2^ > 2σ(*F* ^2^)], *wR*(*F* ^2^), *S*	0.021, 0.057, 1.08	0.024, 0.052, 1.04
No. of reflections	1917	10744
No. of parameters	139	758
H-atom treatment	H-atom parameters constrained	H-atom parameters constrained
Δρ_max_, Δρ_min_ (e Å^−3^)	0.30, −0.34	0.27, −0.25
Absolute structure	–	Flack *x* determined using 4178 quotients [(*I* ^+^)−(*I* ^−^)]/[(*I* ^+^)+(*I* ^−^)] (Parsons *et al*, 2013 ▸)
Absolute structure parameter	–	0.003 (3)

Computer programs: *APEX3* and *SAINT* (Bruker 2016 ▸), *SHELXS97* (Sheldrick 2008 ▸), *SHELXL2014* (Sheldrick, 2015 ▸), *OLEX2* (Dolomanov *et al.* 2009 ▸) and *publCIF* (Westrip 2010 ▸).

## supplementary crystallographic information

### catena-Poly[[tetrakis(pyridine-κN)iron(II)]-µ-sulfato-κ2O:O'] (1) Crystal data

**Table:**

[Fe(SO_4_)(C_5_H_5_N)_4_]	*F*(000) = 968
*M**_r_* = 468.31	*D*_x_ = 1.548 Mg m^−^^3^
Monoclinic, *I*2/*a*	Mo *K*α radiation, λ = 0.71073 Å
*a* = 11.8259 (10) Å	Cell parameters from 9914 reflections
*b* = 10.0847 (9) Å	θ = 3.3–25.7°
*c* = 17.264 (2) Å	µ = 0.89 mm^−^^1^
β = 102.569 (2)°	*T* = 200 K
*V* = 2009.6 (3) Å^3^	Block, yellow
*Z* = 4	0.28 × 0.20 × 0.20 mm

### catena-Poly[[tetrakis(pyridine-κN)iron(II)]-µ-sulfato-κ2O:O'] (1) Data collection

**Table:**

Bruker D8 Venture CMOS diffractometer	1760 reflections with *I* > 2σ(*I*)
φ and ω scans	*R*_int_ = 0.029
Absorption correction: multi-scan (SADABS; Bruker, 2016)	θ_max_ = 25.8°, θ_min_ = 3.4°
*T*_min_ = 0.397, *T*_max_ = 0.429	*h* = −14→14
25476 measured reflections	*k* = −12→12
1917 independent reflections	*l* = −21→21

### catena-Poly[[tetrakis(pyridine-κN)iron(II)]-µ-sulfato-κ2O:O'] (1) Refinement

**Table:**

Refinement on *F*^2^	Hydrogen site location: inferred from neighbouring sites
Least-squares matrix: full	H-atom parameters constrained
*R*[*F*^2^ > 2σ(*F*^2^)] = 0.021	*w* = 1/[σ^2^(*F*_o_^2^) + (0.0262*P*)^2^ + 2.244*P*] where *P* = (*F*_o_^2^ + 2*F*_c_^2^)/3
*wR*(*F*^2^) = 0.057	(Δ/σ)_max_ < 0.001
*S* = 1.08	Δρ_max_ = 0.30 e Å^−^^3^
1917 reflections	Δρ_min_ = −0.34 e Å^−^^3^
139 parameters	Extinction correction: SHELXL, Fc^*^=kFc[1+0.001xFc^2^λ^3^/sin(2θ)]^-1/4^
0 restraints	Extinction coefficient: 0.0097 (5)

### catena-Poly[[tetrakis(pyridine-κN)iron(II)]-µ-sulfato-κ2O:O'] (1) Special details

**Table:**

Geometry. All esds (except the esd in the dihedral angle between two l.s. planes) are estimated using the full covariance matrix. The cell esds are taken into account individually in the estimation of esds in distances, angles and torsion angles; correlations between esds in cell parameters are only used when they are defined by crystal symmetry. An approximate (isotropic) treatment of cell esds is used for estimating esds involving l.s. planes.

### catena-Poly[[tetrakis(pyridine-κN)iron(II)]-µ-sulfato-κ2O:O'] (1) Fractional atomic coordinates and isotropic or equivalent isotropic displacement parameters (Å^2^)

**Table:**

	*x*	*y*	*z*	*U*_iso_*/*U*_eq_	
Fe1	0.0000	0.0000	0.0000	0.01564 (11)	
S1	−0.2500	0.18552 (4)	0.0000	0.01372 (13)	
O1	−0.15276 (9)	0.09746 (12)	−0.00709 (6)	0.0304 (3)	
O2	−0.21834 (10)	0.26521 (11)	0.07151 (6)	0.0312 (3)	
N1	0.09921 (11)	0.18297 (13)	0.04513 (7)	0.0243 (3)	
N2	−0.01093 (10)	0.05257 (12)	−0.12846 (7)	0.0199 (3)	
C1	0.07094 (15)	0.30220 (16)	0.01393 (10)	0.0325 (4)	
H1	0.0058	0.3090	−0.0292	0.039*	
C2	0.13082 (16)	0.41650 (18)	0.04065 (12)	0.0402 (4)	
H2	0.1083	0.4992	0.0157	0.048*	
C3	0.22374 (16)	0.40865 (19)	0.10405 (12)	0.0420 (5)	
H3	0.2659	0.4859	0.1244	0.050*	
C4	0.25409 (16)	0.2869 (2)	0.13723 (12)	0.0475 (5)	
H4	0.3179	0.2783	0.1811	0.057*	
C5	0.19076 (15)	0.17686 (19)	0.10608 (11)	0.0379 (4)	
H5	0.2133	0.0927	0.1291	0.045*	
C6	0.05695 (14)	−0.00921 (16)	−0.16947 (9)	0.0275 (3)	
H6	0.1106	−0.0731	−0.1428	0.033*	
C7	0.05294 (16)	0.01520 (18)	−0.24882 (10)	0.0345 (4)	
H7	0.1040	−0.0299	−0.2754	0.041*	
C8	−0.02595 (14)	0.10558 (17)	−0.28899 (9)	0.0294 (4)	
H8	−0.0300	0.1247	−0.3434	0.035*	
C9	−0.09860 (13)	0.16721 (16)	−0.24801 (9)	0.0272 (3)	
H9	−0.1554	0.2283	−0.2742	0.033*	
C10	−0.08829 (13)	0.13950 (16)	−0.16846 (9)	0.0246 (3)	
H10	−0.1382	0.1840	−0.1407	0.030*	

### catena-Poly[[tetrakis(pyridine-κN)iron(II)]-µ-sulfato-κ2O:O'] (1) Atomic displacement parameters (Å^2^)

**Table:**

	*U*^11^	*U*^22^	*U*^33^	*U*^12^	*U*^13^	*U*^23^
Fe1	0.01379 (16)	0.01886 (17)	0.01416 (16)	0.00346 (10)	0.00276 (10)	0.00193 (10)
S1	0.0124 (2)	0.0146 (2)	0.0134 (2)	0.000	0.00103 (16)	0.000
O1	0.0231 (5)	0.0433 (7)	0.0258 (6)	0.0165 (5)	0.0076 (4)	0.0056 (5)
O2	0.0420 (7)	0.0278 (6)	0.0226 (6)	−0.0088 (5)	0.0044 (5)	−0.0103 (5)
N1	0.0237 (6)	0.0255 (7)	0.0239 (6)	−0.0013 (5)	0.0055 (5)	−0.0004 (5)
N2	0.0197 (6)	0.0235 (6)	0.0161 (6)	0.0005 (5)	0.0028 (5)	0.0025 (5)
C1	0.0359 (9)	0.0277 (8)	0.0322 (9)	0.0002 (7)	0.0041 (7)	0.0004 (7)
C2	0.0438 (10)	0.0269 (9)	0.0521 (11)	−0.0027 (8)	0.0153 (9)	−0.0021 (8)
C3	0.0366 (10)	0.0388 (10)	0.0535 (11)	−0.0148 (8)	0.0160 (9)	−0.0156 (9)
C4	0.0338 (9)	0.0563 (12)	0.0461 (11)	−0.0157 (9)	−0.0049 (8)	−0.0033 (10)
C5	0.0300 (9)	0.0379 (10)	0.0405 (10)	−0.0064 (7)	−0.0036 (7)	0.0059 (8)
C6	0.0299 (8)	0.0322 (9)	0.0206 (8)	0.0098 (6)	0.0058 (6)	0.0053 (6)
C7	0.0424 (10)	0.0416 (10)	0.0228 (8)	0.0132 (8)	0.0142 (7)	0.0034 (7)
C8	0.0350 (8)	0.0367 (9)	0.0161 (7)	0.0005 (7)	0.0049 (6)	0.0061 (6)
C9	0.0251 (7)	0.0326 (8)	0.0221 (8)	0.0038 (6)	0.0013 (6)	0.0087 (6)
C10	0.0231 (7)	0.0298 (8)	0.0213 (7)	0.0042 (6)	0.0057 (6)	0.0038 (6)

### catena-Poly[[tetrakis(pyridine-κN)iron(II)]-µ-sulfato-κ2O:O'] (1) Geometric parameters (Å, º)

**Table:**

Fe1—O1	2.0367 (10)	C2—H2	0.9500
Fe1—O1^i^	2.0367 (10)	C2—C3	1.374 (3)
Fe1—N1	2.2339 (13)	C3—H3	0.9500
Fe1—N1^i^	2.2339 (13)	C3—C4	1.369 (3)
Fe1—N2^i^	2.2564 (12)	C4—H4	0.9500
Fe1—N2	2.2563 (12)	C4—C5	1.381 (3)
S1—O1	1.4790 (10)	C5—H5	0.9500
S1—O1^ii^	1.4790 (10)	C6—H6	0.9500
S1—O2^ii^	1.4522 (10)	C6—C7	1.382 (2)
S1—O2	1.4522 (10)	C7—H7	0.9500
N1—C1	1.330 (2)	C7—C8	1.379 (2)
N1—C5	1.337 (2)	C8—H8	0.9500
N2—C6	1.335 (2)	C8—C9	1.375 (2)
N2—C10	1.3445 (19)	C9—H9	0.9500
C1—H1	0.9500	C9—C10	1.381 (2)
C1—C2	1.379 (2)	C10—H10	0.9500
			
O1—Fe1—O1^i^	180.0	N1—C1—C2	123.70 (16)
O1—Fe1—N1	90.80 (5)	C2—C1—H1	118.2
O1—Fe1—N1^i^	89.20 (5)	C1—C2—H2	120.6
O1^i^—Fe1—N1	89.20 (5)	C3—C2—C1	118.81 (17)
O1^i^—Fe1—N1^i^	90.80 (5)	C3—C2—H2	120.6
O1^i^—Fe1—N2	91.88 (4)	C2—C3—H3	120.8
O1^i^—Fe1—N2^i^	88.12 (4)	C4—C3—C2	118.44 (17)
O1—Fe1—N2^i^	91.88 (4)	C4—C3—H3	120.8
O1—Fe1—N2	88.12 (4)	C3—C4—H4	120.4
N1^i^—Fe1—N1	180.0	C3—C4—C5	119.16 (17)
N1—Fe1—N2	93.56 (4)	C5—C4—H4	120.4
N1—Fe1—N2^i^	86.44 (4)	N1—C5—C4	123.17 (17)
N1^i^—Fe1—N2^i^	93.56 (4)	N1—C5—H5	118.4
N1^i^—Fe1—N2	86.44 (4)	C4—C5—H5	118.4
N2—Fe1—N2^i^	180.0	N2—C6—H6	118.4
O1^ii^—S1—O1	106.19 (10)	N2—C6—C7	123.21 (14)
O2^ii^—S1—O1^ii^	109.96 (6)	C7—C6—H6	118.4
O2—S1—O1^ii^	108.86 (6)	C6—C7—H7	120.3
O2^ii^—S1—O1	108.86 (6)	C8—C7—C6	119.32 (15)
O2—S1—O1	109.96 (6)	C8—C7—H7	120.3
O2—S1—O2^ii^	112.81 (10)	C7—C8—H8	121.0
S1—O1—Fe1	168.60 (8)	C9—C8—C7	118.06 (14)
C1—N1—Fe1	122.61 (10)	C9—C8—H8	121.0
C1—N1—C5	116.71 (14)	C8—C9—H9	120.3
C5—N1—Fe1	120.69 (11)	C8—C9—C10	119.39 (14)
C6—N2—Fe1	119.95 (10)	C10—C9—H9	120.3
C6—N2—C10	116.91 (12)	N2—C10—C9	123.08 (14)
C10—N2—Fe1	123.03 (10)	N2—C10—H10	118.5
N1—C1—H1	118.2	C9—C10—H10	118.5
			
Fe1—N1—C1—C2	−179.92 (13)	C1—C2—C3—C4	1.1 (3)
Fe1—N1—C5—C4	−178.92 (15)	C2—C3—C4—C5	0.0 (3)
Fe1—N2—C6—C7	178.23 (13)	C3—C4—C5—N1	−0.9 (3)
Fe1—N2—C10—C9	−176.94 (12)	C5—N1—C1—C2	0.5 (3)
O1^ii^—S1—O1—Fe1	−132.1 (4)	C6—N2—C10—C9	−0.7 (2)
O2^ii^—S1—O1—Fe1	109.5 (4)	C6—C7—C8—C9	−0.6 (3)
O2—S1—O1—Fe1	−14.5 (4)	C7—C8—C9—C10	1.7 (2)
N1—C1—C2—C3	−1.4 (3)	C8—C9—C10—N2	−1.0 (2)
N2—C6—C7—C8	−1.3 (3)	C10—N2—C6—C7	1.9 (2)
C1—N1—C5—C4	0.7 (3)		

Symmetry codes: (i) −*x*, −*y*, −*z*; (ii) −*x*−1/2, *y*, −*z*.

### catena-Poly[[tetrakis(pyridine-κN)iron(II)]-µ-sulfato-κ2O:O'] (1) Hydrogen-bond geometry (Å, º)

**Table:**

*D*—H···*A*	*D*—H	H···*A*	*D*···*A*	*D*—H···*A*
C6—H6···O2^i^	0.95	2.49	3.4296 (19)	169
C10—H10···O2^ii^	0.95	2.42	3.3621 (19)	171

Symmetry codes: (i) −*x*, −*y*, −*z*; (ii) −*x*−1/2, *y*, −*z*.

### catena-Poly[[tetrakis(pyridine-κN)cobalt(II)]-µ-sulfato-κ2O:O'-[tetrakis(pyridine-κN)cobalt(II)]-µ-sulfato-κ3O,O':O''-[tris(pyridine-κN)cobalt(II)]-µ-sulfato-κ2O:O'] (2) Crystal data

**Table:**

[Co_3_(SO_4_)_3_(C_5_H_5_N)_11_]	*D*_x_ = 1.571 Mg m^−^^3^
*M**_r_* = 1335.07	Mo *K*α radiation, λ = 0.71073 Å
Orthorhombic, *P*2_1_2_1_2_1_	Cell parameters from 9746 reflections
*a* = 9.4583 (5) Å	θ = 3.1–25.7°
*b* = 18.0344 (12) Å	µ = 1.06 mm^−^^1^
*c* = 33.088 (2) Å	*T* = 200 K
*V* = 5644.0 (6) Å^3^	Block, pink
*Z* = 4	0.24 × 0.22 × 0.20 mm
*F*(000) = 2748	

### catena-Poly[[tetrakis(pyridine-κN)cobalt(II)]-µ-sulfato-κ2O:O'-[tetrakis(pyridine-κN)cobalt(II)]-µ-sulfato-κ3O,O':O''-[tris(pyridine-κN)cobalt(II)]-µ-sulfato-κ2O:O'] (2) Data collection

**Table:**

Bruker D8 Venture CMOS diffractometer	9925 reflections with *I* > 2σ(*I*)
φ and ω scans	*R*_int_ = 0.037
Absorption correction: multi-scan (SADABS; Bruker, 2016)	θ_max_ = 25.8°, θ_min_ = 3.1°
*T*_min_ = 0.394, *T*_max_ = 0.429	*h* = −11→11
80759 measured reflections	*k* = −21→22
10744 independent reflections	*l* = −40→40

### catena-Poly[[tetrakis(pyridine-κN)cobalt(II)]-µ-sulfato-κ2O:O'-[tetrakis(pyridine-κN)cobalt(II)]-µ-sulfato-κ3O,O':O''-[tris(pyridine-κN)cobalt(II)]-µ-sulfato-κ2O:O'] (2) Refinement

**Table:**

Refinement on *F*^2^	H-atom parameters constrained
Least-squares matrix: full	*w* = 1/[σ^2^(*F*_o_^2^) + (0.022*P*)^2^ + 1.8031*P*] where *P* = (*F*_o_^2^ + 2*F*_c_^2^)/3
*R*[*F*^2^ > 2σ(*F*^2^)] = 0.024	(Δ/σ)_max_ = 0.002
*wR*(*F*^2^) = 0.052	Δρ_max_ = 0.27 e Å^−^^3^
*S* = 1.04	Δρ_min_ = −0.25 e Å^−^^3^
10744 reflections	Extinction correction: SHELXL, Fc^*^=kFc[1+0.001xFc^2^λ^3^/sin(2θ)]^-1/4^
758 parameters	Extinction coefficient: 0.00161 (11)
0 restraints	Absolute structure: Flack *x* determined using 4178 quotients [(*I*^+^)-(*I*^-^)]/[(*I*^+^)+(*I*^-^)] (Parsons *et al*, 2013)
Primary atom site location: structure-invariant direct methods	Absolute structure parameter: 0.003 (3)
Hydrogen site location: inferred from neighbouring sites	

### catena-Poly[[tetrakis(pyridine-κN)cobalt(II)]-µ-sulfato-κ2O:O'-[tetrakis(pyridine-κN)cobalt(II)]-µ-sulfato-κ3O,O':O''-[tris(pyridine-κN)cobalt(II)]-µ-sulfato-κ2O:O'] (2) Special details

**Table:**

Geometry. All esds (except the esd in the dihedral angle between two l.s. planes) are estimated using the full covariance matrix. The cell esds are taken into account individually in the estimation of esds in distances, angles and torsion angles; correlations between esds in cell parameters are only used when they are defined by crystal symmetry. An approximate (isotropic) treatment of cell esds is used for estimating esds involving l.s. planes.

### catena-Poly[[tetrakis(pyridine-κN)cobalt(II)]-µ-sulfato-κ2O:O'-[tetrakis(pyridine-κN)cobalt(II)]-µ-sulfato-κ3O,O':O''-[tris(pyridine-κN)cobalt(II)]-µ-sulfato-κ2O:O'] (2) Fractional atomic coordinates and isotropic or equivalent isotropic displacement parameters (Å^2^)

**Table:**

	*x*	*y*	*z*	*U*_iso_*/*U*_eq_	
Co1	0.16718 (4)	0.54403 (2)	0.83580 (2)	0.01623 (9)	
Co2	0.35870 (4)	0.54710 (2)	0.66144 (2)	0.01621 (9)	
Co3	0.59070 (4)	0.50833 (2)	0.49027 (2)	0.01955 (10)	
S1	0.03819 (7)	0.45007 (4)	0.91956 (2)	0.01798 (15)	
S2	0.29926 (8)	0.63760 (4)	0.75389 (2)	0.01700 (15)	
S3	0.46950 (8)	0.44201 (4)	0.57942 (2)	0.01900 (16)	
O1	0.1088 (2)	0.47648 (12)	0.95686 (5)	0.0251 (5)	
O2	0.0608 (2)	0.50429 (12)	0.88685 (6)	0.0285 (5)	
O3	0.0860 (3)	0.37748 (12)	0.90752 (7)	0.0370 (6)	
O4	−0.1154 (2)	0.44982 (13)	0.93000 (6)	0.0272 (5)	
O5	0.2544 (2)	0.58060 (13)	0.78285 (7)	0.0338 (6)	
O6	0.3026 (2)	0.60190 (13)	0.71364 (6)	0.0301 (5)	
O7	0.4410 (3)	0.66160 (15)	0.76463 (7)	0.0438 (7)	
O8	0.2016 (3)	0.69844 (14)	0.75349 (8)	0.0482 (7)	
O9	0.4003 (2)	0.49617 (11)	0.60684 (5)	0.0240 (5)	
O10	0.4761 (2)	0.47822 (12)	0.53920 (6)	0.0319 (5)	
O11	0.6112 (2)	0.42540 (12)	0.59372 (7)	0.0322 (5)	
O12	0.3821 (3)	0.37572 (12)	0.57694 (7)	0.0378 (6)	
N1	0.2390 (3)	0.63476 (14)	0.87321 (7)	0.0200 (5)	
N2	0.3685 (2)	0.49064 (14)	0.84860 (7)	0.0215 (5)	
N3	0.1051 (2)	0.44662 (13)	0.80158 (6)	0.0195 (5)	
N4	−0.0243 (3)	0.60258 (14)	0.82167 (7)	0.0239 (6)	
N5	0.4720 (2)	0.46432 (14)	0.69517 (7)	0.0214 (5)	
N6	0.5501 (3)	0.61413 (14)	0.65736 (8)	0.0241 (6)	
N7	0.2377 (3)	0.62948 (14)	0.62718 (7)	0.0204 (5)	
N8	0.1580 (2)	0.48519 (14)	0.66427 (7)	0.0221 (5)	
N9	0.6037 (3)	0.39214 (14)	0.47104 (7)	0.0261 (6)	
N10	0.8148 (3)	0.50203 (14)	0.50131 (6)	0.0237 (6)	
N11	0.5986 (3)	0.62212 (14)	0.51336 (7)	0.0259 (6)	
C1	0.2806 (3)	0.62099 (18)	0.91129 (9)	0.0277 (7)	
H1	0.2668	0.5726	0.9219	0.033*	
C2	0.3421 (4)	0.67357 (19)	0.93563 (10)	0.0367 (9)	
H2	0.3717	0.6612	0.9622	0.044*	
C3	0.3605 (4)	0.74465 (18)	0.92107 (10)	0.0353 (8)	
H3	0.4024	0.7820	0.9374	0.042*	
C4	0.3165 (4)	0.76014 (18)	0.88237 (10)	0.0314 (8)	
H4	0.3270	0.8086	0.8716	0.038*	
C5	0.2569 (3)	0.70427 (17)	0.85935 (9)	0.0251 (7)	
H5	0.2273	0.7155	0.8326	0.030*	
C6	0.3782 (3)	0.41984 (17)	0.86044 (9)	0.0247 (7)	
H6	0.2941	0.3913	0.8627	0.030*	
C7	0.5059 (3)	0.38681 (19)	0.86950 (10)	0.0327 (8)	
H7	0.5095	0.3359	0.8767	0.039*	
C8	0.6277 (3)	0.4281 (2)	0.86801 (9)	0.0316 (8)	
H8	0.7161	0.4069	0.8753	0.038*	
C9	0.6188 (3)	0.50081 (19)	0.85578 (10)	0.0335 (8)	
H9	0.7012	0.5308	0.8543	0.040*	
C10	0.4884 (3)	0.52946 (18)	0.84573 (10)	0.0291 (8)	
H10	0.4836	0.5792	0.8363	0.035*	
C11	−0.0126 (3)	0.40794 (18)	0.80984 (9)	0.0263 (7)	
H11	−0.0724	0.4248	0.8310	0.032*	
C12	−0.0507 (4)	0.34498 (19)	0.78909 (10)	0.0321 (8)	
H12	−0.1350	0.3193	0.7960	0.038*	
C13	0.0338 (4)	0.31956 (18)	0.75843 (10)	0.0337 (8)	
H13	0.0092	0.2762	0.7437	0.040*	
C14	0.1552 (4)	0.35841 (18)	0.74950 (10)	0.0346 (8)	
H14	0.2164	0.3420	0.7286	0.041*	
C15	0.1867 (3)	0.42136 (18)	0.77132 (9)	0.0275 (7)	
H15	0.2700	0.4481	0.7647	0.033*	
C16	−0.0909 (3)	0.64654 (18)	0.84787 (10)	0.0339 (8)	
H16	−0.0548	0.6504	0.8746	0.041*	
C17	−0.2093 (4)	0.6865 (2)	0.83787 (15)	0.0514 (11)	
H17	−0.2526	0.7182	0.8572	0.062*	
C18	−0.2643 (4)	0.6801 (2)	0.79988 (15)	0.0534 (12)	
H18	−0.3472	0.7065	0.7925	0.064*	
C19	−0.1976 (4)	0.6347 (2)	0.77261 (12)	0.0461 (10)	
H19	−0.2341	0.6289	0.7461	0.055*	
C20	−0.0775 (4)	0.59795 (19)	0.78418 (10)	0.0332 (8)	
H20	−0.0301	0.5679	0.7649	0.040*	
C21	0.5379 (3)	0.48265 (18)	0.72972 (9)	0.0272 (7)	
H21	0.5375	0.5331	0.7380	0.033*	
C22	0.6060 (4)	0.4314 (2)	0.75364 (10)	0.0378 (9)	
H22	0.6533	0.4466	0.7776	0.045*	
C23	0.6049 (4)	0.3573 (2)	0.74234 (10)	0.0392 (9)	
H23	0.6494	0.3208	0.7586	0.047*	
C24	0.5380 (3)	0.33802 (19)	0.70710 (10)	0.0299 (7)	
H24	0.5356	0.2877	0.6986	0.036*	
C25	0.4744 (3)	0.39224 (17)	0.68426 (9)	0.0246 (7)	
H25	0.4301	0.3783	0.6596	0.029*	
C26	0.6599 (4)	0.5926 (2)	0.63510 (12)	0.0460 (10)	
H26	0.6565	0.5456	0.6221	0.055*	
C27	0.7784 (4)	0.6362 (3)	0.63026 (17)	0.0759 (16)	
H27	0.8545	0.6194	0.6140	0.091*	
C28	0.7863 (4)	0.7039 (3)	0.64894 (16)	0.0666 (14)	
H28	0.8664	0.7350	0.6454	0.080*	
C29	0.6767 (4)	0.7253 (2)	0.67265 (11)	0.0404 (9)	
H29	0.6801	0.7712	0.6868	0.048*	
C30	0.5605 (4)	0.67976 (18)	0.67597 (9)	0.0292 (8)	
H30	0.4838	0.6957	0.6923	0.035*	
C31	0.2091 (3)	0.69806 (18)	0.64044 (10)	0.0275 (7)	
H31	0.2413	0.7119	0.6666	0.033*	
C32	0.1354 (4)	0.74964 (18)	0.61799 (10)	0.0305 (8)	
H32	0.1195	0.7981	0.6283	0.037*	
C33	0.0852 (4)	0.72998 (19)	0.58052 (10)	0.0347 (8)	
H33	0.0337	0.7644	0.5645	0.042*	
C34	0.1113 (4)	0.65970 (19)	0.56676 (10)	0.0365 (9)	
H34	0.0770	0.6445	0.5411	0.044*	
C35	0.1877 (3)	0.61102 (17)	0.59052 (9)	0.0273 (7)	
H35	0.2056	0.5625	0.5805	0.033*	
C36	0.0511 (3)	0.5147 (2)	0.68519 (10)	0.0351 (8)	
H36	0.0665	0.5600	0.6991	0.042*	
C37	−0.0802 (4)	0.4823 (2)	0.68751 (12)	0.0514 (11)	
H37	−0.1537	0.5053	0.7025	0.062*	
C38	−0.1037 (4)	0.4168 (3)	0.66800 (13)	0.0596 (12)	
H38	−0.1936	0.3935	0.6693	0.072*	
C39	0.0050 (4)	0.3849 (2)	0.64635 (11)	0.0459 (10)	
H39	−0.0083	0.3391	0.6327	0.055*	
C40	0.1339 (4)	0.42110 (18)	0.64486 (9)	0.0287 (7)	
H40	0.2082	0.3997	0.6295	0.034*	
C41	0.4994 (4)	0.34518 (19)	0.47977 (10)	0.0345 (8)	
H41	0.4166	0.3641	0.4924	0.041*	
C42	0.5062 (5)	0.2702 (2)	0.47129 (11)	0.0497 (11)	
H42	0.4282	0.2388	0.4771	0.060*	
C43	0.6272 (5)	0.2417 (2)	0.45436 (11)	0.0486 (11)	
H43	0.6355	0.1901	0.4491	0.058*	
C44	0.7343 (4)	0.2886 (2)	0.44538 (12)	0.0508 (11)	
H44	0.8193	0.2704	0.4337	0.061*	
C45	0.7180 (4)	0.3632 (2)	0.45343 (11)	0.0404 (9)	
H45	0.7925	0.3958	0.4460	0.048*	
C46	0.8681 (3)	0.47053 (18)	0.53457 (9)	0.0284 (7)	
H46	0.8053	0.4534	0.5549	0.034*	
C47	1.0131 (4)	0.4622 (2)	0.54019 (10)	0.0342 (8)	
H47	1.0481	0.4399	0.5642	0.041*	
C48	1.1055 (3)	0.48622 (18)	0.51078 (10)	0.0327 (7)	
H48	1.2046	0.4803	0.5141	0.039*	
C49	1.0512 (3)	0.51890 (18)	0.47664 (10)	0.0313 (8)	
H49	1.1119	0.5365	0.4559	0.038*	
C50	0.9062 (3)	0.52552 (18)	0.47324 (9)	0.0284 (7)	
H50	0.8692	0.5481	0.4496	0.034*	
C51	0.5073 (4)	0.6448 (2)	0.54108 (12)	0.0498 (11)	
H51	0.4341	0.6119	0.5491	0.060*	
C52	0.5136 (6)	0.7137 (2)	0.55876 (15)	0.0772 (17)	
H52	0.4435	0.7285	0.5777	0.093*	
C53	0.6227 (6)	0.7613 (2)	0.54885 (12)	0.0583 (13)	
H53	0.6318	0.8082	0.5616	0.070*	
C54	0.7162 (4)	0.7388 (2)	0.52031 (12)	0.0491 (10)	
H54	0.7921	0.7700	0.5123	0.059*	
C55	0.6991 (4)	0.6698 (2)	0.50314 (12)	0.0434 (10)	
H55	0.7637	0.6555	0.4826	0.052*	

### catena-Poly[[tetrakis(pyridine-κN)cobalt(II)]-µ-sulfato-κ2O:O'-[tetrakis(pyridine-κN)cobalt(II)]-µ-sulfato-κ3O,O':O''-[tris(pyridine-κN)cobalt(II)]-µ-sulfato-κ2O:O'] (2) Atomic displacement parameters (Å^2^)

**Table:**

	*U*^11^	*U*^22^	*U*^33^	*U*^12^	*U*^13^	*U*^23^
Co1	0.01839 (19)	0.01621 (19)	0.01408 (18)	−0.00176 (16)	−0.00010 (15)	0.00116 (16)
Co2	0.01785 (19)	0.01691 (19)	0.01386 (18)	−0.00072 (16)	0.00057 (15)	−0.00016 (16)
Co3	0.0178 (2)	0.0255 (2)	0.01525 (18)	0.00081 (17)	−0.00025 (16)	−0.00245 (17)
S1	0.0193 (3)	0.0192 (4)	0.0154 (3)	−0.0044 (3)	0.0025 (3)	0.0003 (3)
S2	0.0219 (4)	0.0145 (4)	0.0146 (3)	−0.0009 (3)	0.0017 (3)	−0.0001 (3)
S3	0.0236 (4)	0.0161 (4)	0.0173 (3)	0.0024 (3)	0.0040 (3)	−0.0007 (3)
O1	0.0216 (11)	0.0362 (13)	0.0174 (10)	−0.0044 (9)	−0.0017 (8)	−0.0007 (9)
O2	0.0389 (13)	0.0287 (12)	0.0178 (10)	−0.0071 (10)	0.0072 (9)	0.0035 (9)
O3	0.0491 (15)	0.0185 (12)	0.0435 (14)	−0.0039 (11)	0.0156 (12)	−0.0039 (10)
O4	0.0182 (10)	0.0429 (13)	0.0206 (10)	−0.0075 (10)	0.0001 (8)	0.0025 (10)
O5	0.0458 (14)	0.0314 (13)	0.0242 (12)	−0.0068 (11)	0.0087 (11)	0.0073 (10)
O6	0.0324 (13)	0.0421 (14)	0.0158 (11)	−0.0048 (11)	0.0037 (10)	−0.0078 (10)
O7	0.0341 (14)	0.0550 (17)	0.0421 (14)	−0.0208 (12)	−0.0071 (11)	−0.0067 (12)
O8	0.0581 (17)	0.0342 (15)	0.0523 (16)	0.0260 (13)	−0.0034 (14)	0.0000 (12)
O9	0.0280 (11)	0.0260 (11)	0.0180 (10)	0.0058 (10)	0.0039 (9)	−0.0049 (9)
O10	0.0434 (14)	0.0348 (13)	0.0175 (10)	0.0042 (11)	0.0066 (10)	0.0029 (9)
O11	0.0267 (12)	0.0316 (13)	0.0382 (13)	0.0100 (10)	0.0007 (10)	0.0059 (10)
O12	0.0412 (14)	0.0225 (12)	0.0498 (15)	−0.0100 (11)	0.0106 (12)	−0.0075 (11)
N1	0.0238 (13)	0.0187 (14)	0.0176 (12)	−0.0009 (11)	−0.0012 (10)	−0.0002 (10)
N2	0.0211 (13)	0.0222 (14)	0.0213 (12)	−0.0010 (11)	−0.0011 (10)	0.0019 (11)
N3	0.0198 (12)	0.0213 (13)	0.0173 (12)	−0.0034 (11)	−0.0004 (10)	0.0018 (10)
N4	0.0220 (13)	0.0260 (14)	0.0237 (13)	0.0003 (11)	−0.0003 (11)	0.0045 (11)
N5	0.0201 (12)	0.0236 (14)	0.0204 (12)	0.0013 (11)	0.0026 (10)	0.0017 (11)
N6	0.0222 (13)	0.0240 (14)	0.0260 (14)	−0.0006 (11)	0.0009 (11)	0.0018 (11)
N7	0.0228 (13)	0.0200 (14)	0.0183 (12)	0.0006 (11)	0.0008 (10)	−0.0013 (11)
N8	0.0206 (12)	0.0282 (13)	0.0176 (12)	−0.0031 (11)	0.0011 (11)	0.0023 (11)
N9	0.0283 (15)	0.0284 (15)	0.0216 (13)	0.0033 (12)	−0.0022 (12)	−0.0044 (11)
N10	0.0212 (13)	0.0293 (15)	0.0206 (13)	0.0012 (12)	−0.0004 (10)	−0.0041 (11)
N11	0.0276 (14)	0.0274 (14)	0.0226 (13)	0.0008 (12)	−0.0024 (12)	−0.0040 (11)
C1	0.0362 (19)	0.0236 (18)	0.0233 (17)	−0.0070 (14)	−0.0050 (14)	0.0038 (13)
C2	0.059 (2)	0.0288 (19)	0.0226 (17)	−0.0110 (18)	−0.0122 (17)	0.0026 (14)
C3	0.047 (2)	0.0271 (18)	0.0312 (18)	−0.0119 (16)	−0.0056 (17)	−0.0073 (15)
C4	0.044 (2)	0.0186 (17)	0.0314 (18)	−0.0058 (15)	−0.0009 (16)	0.0024 (14)
C5	0.0349 (19)	0.0214 (17)	0.0188 (15)	−0.0002 (14)	−0.0005 (14)	0.0032 (13)
C6	0.0250 (17)	0.0257 (17)	0.0233 (16)	−0.0018 (14)	0.0011 (13)	0.0027 (13)
C7	0.033 (2)	0.033 (2)	0.0329 (18)	0.0071 (15)	−0.0017 (15)	0.0094 (15)
C8	0.0220 (17)	0.049 (2)	0.0235 (16)	0.0096 (15)	−0.0019 (13)	−0.0001 (15)
C9	0.0239 (17)	0.038 (2)	0.0389 (18)	−0.0030 (16)	0.0005 (14)	−0.0079 (17)
C10	0.0228 (16)	0.0256 (18)	0.0389 (19)	−0.0038 (13)	0.0001 (14)	−0.0007 (14)
C11	0.0250 (17)	0.0346 (19)	0.0194 (16)	−0.0085 (15)	0.0023 (13)	0.0023 (14)
C12	0.0340 (19)	0.0356 (19)	0.0266 (17)	−0.0197 (16)	−0.0054 (15)	0.0062 (15)
C13	0.046 (2)	0.0236 (18)	0.0316 (19)	−0.0086 (16)	−0.0084 (16)	−0.0037 (15)
C14	0.036 (2)	0.0309 (19)	0.0365 (19)	0.0010 (16)	0.0083 (16)	−0.0098 (16)
C15	0.0245 (17)	0.0280 (18)	0.0300 (17)	−0.0055 (14)	0.0056 (14)	−0.0024 (14)
C16	0.0283 (17)	0.0319 (19)	0.041 (2)	0.0016 (15)	−0.0030 (16)	−0.0074 (15)
C17	0.035 (2)	0.041 (2)	0.078 (3)	0.0103 (18)	0.000 (2)	−0.013 (2)
C18	0.029 (2)	0.035 (2)	0.096 (4)	0.0067 (18)	−0.020 (2)	0.007 (2)
C19	0.040 (2)	0.044 (2)	0.054 (2)	−0.0050 (19)	−0.0229 (19)	0.011 (2)
C20	0.036 (2)	0.035 (2)	0.0289 (18)	0.0015 (16)	−0.0069 (16)	0.0031 (15)
C21	0.0260 (16)	0.0309 (18)	0.0247 (16)	0.0031 (14)	−0.0028 (13)	−0.0050 (14)
C22	0.037 (2)	0.044 (2)	0.0316 (18)	0.0133 (17)	−0.0107 (16)	−0.0010 (16)
C23	0.036 (2)	0.043 (2)	0.038 (2)	0.0116 (17)	−0.0038 (17)	0.0130 (17)
C24	0.0290 (17)	0.0253 (17)	0.0356 (19)	0.0024 (14)	0.0039 (15)	0.0066 (14)
C25	0.0251 (16)	0.0220 (17)	0.0266 (17)	−0.0004 (14)	0.0005 (14)	0.0012 (13)
C26	0.029 (2)	0.040 (2)	0.070 (3)	−0.0032 (17)	0.0128 (19)	−0.016 (2)
C27	0.029 (2)	0.071 (3)	0.128 (5)	−0.017 (2)	0.033 (3)	−0.032 (3)
C28	0.032 (2)	0.057 (3)	0.111 (4)	−0.026 (2)	0.011 (2)	−0.016 (3)
C29	0.040 (2)	0.033 (2)	0.048 (2)	−0.0149 (17)	−0.0048 (18)	0.0003 (17)
C30	0.0346 (19)	0.0310 (19)	0.0219 (16)	−0.0068 (15)	0.0025 (14)	0.0027 (14)
C31	0.0317 (18)	0.0255 (18)	0.0252 (17)	0.0037 (14)	−0.0032 (14)	−0.0040 (14)
C32	0.0358 (19)	0.0222 (17)	0.0336 (18)	0.0070 (15)	0.0044 (15)	−0.0003 (14)
C33	0.040 (2)	0.0322 (19)	0.0314 (18)	0.0139 (16)	−0.0049 (16)	0.0061 (15)
C34	0.048 (2)	0.037 (2)	0.0249 (17)	0.0148 (18)	−0.0113 (16)	−0.0048 (15)
C35	0.0346 (18)	0.0221 (17)	0.0250 (16)	0.0038 (14)	−0.0043 (14)	−0.0048 (13)
C36	0.0277 (18)	0.043 (2)	0.0343 (18)	−0.0035 (16)	0.0055 (14)	−0.0097 (16)
C37	0.0241 (18)	0.078 (3)	0.053 (2)	−0.013 (2)	0.0101 (17)	−0.024 (2)
C38	0.034 (2)	0.086 (3)	0.059 (3)	−0.032 (2)	0.011 (2)	−0.024 (2)
C39	0.044 (2)	0.054 (3)	0.039 (2)	−0.026 (2)	0.0069 (18)	−0.0106 (18)
C40	0.0304 (18)	0.0351 (19)	0.0204 (15)	−0.0062 (15)	−0.0003 (13)	−0.0022 (14)
C41	0.041 (2)	0.035 (2)	0.0282 (18)	−0.0023 (16)	0.0024 (15)	−0.0058 (15)
C42	0.074 (3)	0.035 (2)	0.040 (2)	−0.013 (2)	0.006 (2)	−0.0027 (17)
C43	0.080 (3)	0.028 (2)	0.038 (2)	0.012 (2)	−0.011 (2)	−0.0060 (17)
C44	0.046 (2)	0.052 (3)	0.054 (3)	0.022 (2)	−0.007 (2)	−0.026 (2)
C45	0.031 (2)	0.043 (2)	0.047 (2)	0.0008 (17)	0.0022 (17)	−0.0170 (18)
C46	0.0286 (17)	0.0326 (19)	0.0240 (16)	−0.0002 (14)	−0.0007 (13)	0.0005 (14)
C47	0.0332 (19)	0.040 (2)	0.0292 (17)	0.0062 (16)	−0.0115 (14)	0.0012 (16)
C48	0.0156 (15)	0.0395 (19)	0.0430 (18)	0.0033 (14)	−0.0039 (15)	−0.0142 (17)
C49	0.0251 (17)	0.0345 (19)	0.0342 (18)	−0.0025 (14)	0.0066 (14)	−0.0082 (15)
C50	0.0247 (16)	0.037 (2)	0.0234 (15)	0.0021 (14)	0.0023 (14)	−0.0006 (13)
C51	0.057 (3)	0.037 (2)	0.055 (2)	−0.006 (2)	0.028 (2)	−0.0115 (19)
C52	0.118 (4)	0.039 (3)	0.075 (3)	−0.012 (3)	0.059 (3)	−0.022 (2)
C53	0.103 (4)	0.029 (2)	0.042 (2)	−0.012 (2)	0.012 (3)	−0.0094 (18)
C54	0.053 (2)	0.033 (2)	0.062 (3)	−0.0096 (18)	0.002 (2)	0.005 (2)
C55	0.045 (2)	0.036 (2)	0.049 (2)	−0.0020 (18)	0.0167 (18)	−0.0037 (17)

### catena-Poly[[tetrakis(pyridine-κN)cobalt(II)]-µ-sulfato-κ2O:O'-[tetrakis(pyridine-κN)cobalt(II)]-µ-sulfato-κ3O,O':O''-[tris(pyridine-κN)cobalt(II)]-µ-sulfato-κ2O:O'] (2) Geometric parameters (Å, º)

**Table:**

Co1—O2	2.0924 (19)	C13—H13	0.9500
Co1—O5	2.046 (2)	C13—C14	1.377 (5)
Co1—N1	2.161 (2)	C14—H14	0.9500
Co1—N2	2.175 (2)	C14—C15	1.378 (4)
Co1—N3	2.171 (2)	C15—H15	0.9500
Co1—N4	2.148 (3)	C16—H16	0.9500
Co2—O6	2.060 (2)	C16—C17	1.372 (5)
Co2—O9	2.0646 (18)	C17—H17	0.9500
Co2—N5	2.150 (2)	C17—C18	1.365 (6)
Co2—N6	2.181 (2)	C18—H18	0.9500
Co2—N7	2.192 (2)	C18—C19	1.372 (6)
Co2—N8	2.204 (2)	C19—H19	0.9500
Co3—S1^i^	2.7428 (8)	C19—C20	1.370 (5)
Co3—O1^i^	2.204 (2)	C20—H20	0.9500
Co3—O4^i^	2.145 (2)	C21—H21	0.9500
Co3—O10	2.022 (2)	C21—C22	1.376 (4)
Co3—N9	2.193 (3)	C22—H22	0.9500
Co3—N10	2.154 (2)	C22—C23	1.388 (5)
Co3—N11	2.191 (2)	C23—H23	0.9500
S1—Co3^ii^	2.7429 (8)	C23—C24	1.371 (5)
S1—O1	1.482 (2)	C24—H24	0.9500
S1—O2	1.474 (2)	C24—C25	1.374 (4)
S1—O3	1.441 (2)	C25—H25	0.9500
S1—O4	1.493 (2)	C26—H26	0.9500
S2—O5	1.468 (2)	C26—C27	1.379 (5)
S2—O6	1.480 (2)	C27—H27	0.9500
S2—O7	1.453 (2)	C27—C28	1.370 (6)
S2—O8	1.435 (2)	C28—H28	0.9500
S3—O9	1.485 (2)	C28—C29	1.356 (6)
S3—O10	1.484 (2)	C29—H29	0.9500
S3—O11	1.453 (2)	C29—C30	1.377 (5)
S3—O12	1.456 (2)	C30—H30	0.9500
O1—Co3^ii^	2.204 (2)	C31—H31	0.9500
O4—Co3^ii^	2.145 (2)	C31—C32	1.380 (4)
N1—C1	1.343 (4)	C32—H32	0.9500
N1—C5	1.346 (4)	C32—C33	1.374 (5)
N2—C6	1.339 (4)	C33—H33	0.9500
N2—C10	1.336 (4)	C33—C34	1.369 (5)
N3—C11	1.342 (4)	C34—H34	0.9500
N3—C15	1.344 (4)	C34—C35	1.383 (4)
N4—C16	1.333 (4)	C35—H35	0.9500
N4—C20	1.341 (4)	C36—H36	0.9500
N5—C21	1.344 (4)	C36—C37	1.374 (5)
N5—C25	1.349 (4)	C37—H37	0.9500
N6—C26	1.331 (4)	C37—C38	1.364 (5)
N6—C30	1.338 (4)	C38—H38	0.9500
N7—C31	1.340 (4)	C38—C39	1.379 (5)
N7—C35	1.344 (4)	C39—H39	0.9500
N8—C36	1.335 (4)	C39—C40	1.383 (5)
N8—C40	1.342 (4)	C40—H40	0.9500
N9—C41	1.332 (4)	C41—H41	0.9500
N9—C45	1.335 (4)	C41—C42	1.383 (5)
N10—C46	1.337 (4)	C42—H42	0.9500
N10—C50	1.338 (4)	C42—C43	1.374 (6)
N11—C51	1.324 (4)	C43—H43	0.9500
N11—C55	1.326 (4)	C43—C44	1.352 (6)
C1—H1	0.9500	C44—H44	0.9500
C1—C2	1.373 (5)	C44—C45	1.380 (5)
C2—H2	0.9500	C45—H45	0.9500
C2—C3	1.380 (5)	C46—H46	0.9500
C3—H3	0.9500	C46—C47	1.391 (5)
C3—C4	1.375 (5)	C47—H47	0.9500
C4—H4	0.9500	C47—C48	1.378 (5)
C4—C5	1.383 (4)	C48—H48	0.9500
C5—H5	0.9500	C48—C49	1.374 (5)
C6—H6	0.9500	C49—H49	0.9500
C6—C7	1.380 (4)	C49—C50	1.381 (4)
C7—H7	0.9500	C50—H50	0.9500
C7—C8	1.372 (5)	C51—H51	0.9500
C8—H8	0.9500	C51—C52	1.375 (5)
C8—C9	1.375 (5)	C52—H52	0.9500
C9—H9	0.9500	C52—C53	1.381 (6)
C9—C10	1.378 (4)	C53—H53	0.9500
C10—H10	0.9500	C53—C54	1.356 (6)
C11—H11	0.9500	C54—H54	0.9500
C11—C12	1.375 (4)	C54—C55	1.377 (5)
C12—H12	0.9500	C55—H55	0.9500
C12—C13	1.370 (5)		
			
O2—Co1—N1	87.02 (9)	C10—C9—H9	120.5
O2—Co1—N2	96.43 (9)	N2—C10—C9	123.1 (3)
O2—Co1—N3	90.79 (8)	N2—C10—H10	118.5
O2—Co1—N4	86.49 (9)	C9—C10—H10	118.5
O5—Co1—O2	174.62 (9)	N3—C11—H11	118.5
O5—Co1—N1	96.87 (9)	N3—C11—C12	123.0 (3)
O5—Co1—N2	87.50 (9)	C12—C11—H11	118.5
O5—Co1—N3	85.60 (9)	C11—C12—H12	120.2
O5—Co1—N4	89.73 (10)	C13—C12—C11	119.6 (3)
N1—Co1—N2	87.06 (9)	C13—C12—H12	120.2
N1—Co1—N3	175.13 (9)	C12—C13—H13	120.8
N3—Co1—N2	88.87 (9)	C12—C13—C14	118.3 (3)
N4—Co1—N1	90.99 (10)	C14—C13—H13	120.8
N4—Co1—N2	176.40 (10)	C13—C14—H14	120.4
N4—Co1—N3	93.21 (9)	C13—C14—C15	119.2 (3)
O6—Co2—O9	175.16 (9)	C15—C14—H14	120.4
O6—Co2—N5	91.52 (9)	N3—C15—C14	123.0 (3)
O6—Co2—N6	90.00 (9)	N3—C15—H15	118.5
O6—Co2—N7	88.49 (9)	C14—C15—H15	118.5
O6—Co2—N8	89.17 (9)	N4—C16—H16	118.6
O9—Co2—N5	92.90 (8)	N4—C16—C17	122.8 (3)
O9—Co2—N6	91.97 (9)	C17—C16—H16	118.6
O9—Co2—N7	87.04 (8)	C16—C17—H17	120.4
O9—Co2—N8	88.61 (8)	C18—C17—C16	119.2 (4)
N5—Co2—N6	90.19 (9)	C18—C17—H17	120.4
N5—Co2—N7	178.34 (9)	C17—C18—H18	120.6
N5—Co2—N8	93.19 (9)	C17—C18—C19	118.8 (4)
N6—Co2—N7	91.48 (9)	C19—C18—H18	120.6
N6—Co2—N8	176.54 (9)	C18—C19—H19	120.5
N7—Co2—N8	85.15 (9)	C20—C19—C18	119.1 (4)
O1^i^—Co3—S1^i^	32.61 (5)	C20—C19—H19	120.5
O4^i^—Co3—S1^i^	32.76 (5)	N4—C20—C19	122.7 (3)
O4^i^—Co3—O1^i^	65.36 (7)	N4—C20—H20	118.7
O4^i^—Co3—N9	93.46 (9)	C19—C20—H20	118.7
O4^i^—Co3—N10	93.97 (8)	N5—C21—H21	118.6
O4^i^—Co3—N11	89.48 (9)	N5—C21—C22	122.8 (3)
O10—Co3—S1^i^	121.20 (7)	C22—C21—H21	118.6
O10—Co3—O1^i^	88.63 (8)	C21—C22—H22	120.4
O10—Co3—O4^i^	153.76 (8)	C21—C22—C23	119.2 (3)
O10—Co3—N9	90.33 (9)	C23—C22—H22	120.4
O10—Co3—N10	112.18 (9)	C22—C23—H23	120.8
O10—Co3—N11	89.46 (9)	C24—C23—C22	118.5 (3)
N9—Co3—S1^i^	92.22 (7)	C24—C23—H23	120.8
N9—Co3—O1^i^	91.22 (9)	C23—C24—H24	120.4
N10—Co3—S1^i^	126.62 (6)	C23—C24—C25	119.3 (3)
N10—Co3—O1^i^	159.09 (8)	C25—C24—H24	120.4
N10—Co3—N9	86.76 (10)	N5—C25—C24	123.1 (3)
N10—Co3—N11	87.52 (9)	N5—C25—H25	118.5
N11—Co3—S1^i^	93.23 (7)	C24—C25—H25	118.5
N11—Co3—O1^i^	95.03 (9)	N6—C26—H26	118.9
N11—Co3—N9	173.74 (10)	N6—C26—C27	122.1 (4)
O1—S1—Co3^ii^	53.26 (8)	C27—C26—H26	118.9
O1—S1—O4	104.28 (11)	C26—C27—H27	120.0
O2—S1—Co3^ii^	120.63 (9)	C28—C27—C26	120.1 (4)
O2—S1—O1	109.44 (12)	C28—C27—H27	120.0
O2—S1—O4	108.25 (13)	C27—C28—H28	120.9
O3—S1—Co3^ii^	128.59 (10)	C29—C28—C27	118.2 (4)
O3—S1—O1	112.38 (14)	C29—C28—H28	120.9
O3—S1—O2	110.73 (13)	C28—C29—H29	120.5
O3—S1—O4	111.51 (14)	C28—C29—C30	119.1 (3)
O4—S1—Co3^ii^	51.03 (8)	C30—C29—H29	120.5
O5—S2—O6	106.80 (14)	N6—C30—C29	123.4 (3)
O7—S2—O5	108.38 (15)	N6—C30—H30	118.3
O7—S2—O6	109.30 (14)	C29—C30—H30	118.3
O8—S2—O5	110.82 (16)	N7—C31—H31	118.4
O8—S2—O6	109.77 (14)	N7—C31—C32	123.2 (3)
O8—S2—O7	111.64 (17)	C32—C31—H31	118.4
O10—S3—O9	106.08 (12)	C31—C32—H32	120.4
O11—S3—O9	110.10 (13)	C33—C32—C31	119.1 (3)
O11—S3—O10	110.11 (13)	C33—C32—H32	120.4
O11—S3—O12	111.91 (14)	C32—C33—H33	120.8
O12—S3—O9	108.91 (13)	C34—C33—C32	118.5 (3)
O12—S3—O10	109.55 (14)	C34—C33—H33	120.8
S1—O1—Co3^ii^	94.13 (10)	C33—C34—H34	120.2
S1—O2—Co1	152.83 (14)	C33—C34—C35	119.5 (3)
S1—O4—Co3^ii^	96.21 (10)	C35—C34—H34	120.2
S2—O5—Co1	154.34 (16)	N7—C35—C34	122.7 (3)
S2—O6—Co2	165.71 (15)	N7—C35—H35	118.7
S3—O9—Co2	155.64 (13)	C34—C35—H35	118.7
S3—O10—Co3	149.17 (15)	N8—C36—H36	118.5
C1—N1—Co1	119.3 (2)	N8—C36—C37	123.0 (3)
C1—N1—C5	117.1 (3)	C37—C36—H36	118.5
C5—N1—Co1	123.35 (19)	C36—C37—H37	120.4
C6—N2—Co1	122.6 (2)	C38—C37—C36	119.2 (4)
C10—N2—Co1	119.8 (2)	C38—C37—H37	120.4
C10—N2—C6	117.5 (3)	C37—C38—H38	120.5
C11—N3—Co1	122.6 (2)	C37—C38—C39	119.0 (3)
C11—N3—C15	116.9 (3)	C39—C38—H38	120.5
C15—N3—Co1	120.5 (2)	C38—C39—H39	120.7
C16—N4—Co1	123.3 (2)	C38—C39—C40	118.6 (3)
C16—N4—C20	117.5 (3)	C40—C39—H39	120.7
C20—N4—Co1	119.2 (2)	N8—C40—C39	122.6 (3)
C21—N5—Co2	120.1 (2)	N8—C40—H40	118.7
C21—N5—C25	117.2 (3)	C39—C40—H40	118.7
C25—N5—Co2	122.6 (2)	N9—C41—H41	118.5
C26—N6—Co2	121.3 (2)	N9—C41—C42	122.9 (3)
C26—N6—C30	117.1 (3)	C42—C41—H41	118.5
C30—N6—Co2	121.5 (2)	C41—C42—H42	120.4
C31—N7—Co2	124.2 (2)	C43—C42—C41	119.1 (4)
C31—N7—C35	117.0 (3)	C43—C42—H42	120.4
C35—N7—Co2	118.9 (2)	C42—C43—H43	120.7
C36—N8—Co2	118.2 (2)	C44—C43—C42	118.7 (4)
C36—N8—C40	117.5 (3)	C44—C43—H43	120.7
C40—N8—Co2	124.2 (2)	C43—C44—H44	120.5
C41—N9—Co3	120.2 (2)	C43—C44—C45	118.9 (4)
C41—N9—C45	116.5 (3)	C45—C44—H44	120.5
C45—N9—Co3	123.1 (2)	N9—C45—C44	123.8 (4)
C46—N10—Co3	122.2 (2)	N9—C45—H45	118.1
C46—N10—C50	117.5 (3)	C44—C45—H45	118.1
C50—N10—Co3	120.11 (19)	N10—C46—H46	119.1
C51—N11—Co3	120.5 (2)	N10—C46—C47	121.9 (3)
C51—N11—C55	116.4 (3)	C47—C46—H46	119.1
C55—N11—Co3	122.9 (2)	C46—C47—H47	120.1
N1—C1—H1	118.4	C48—C47—C46	119.8 (3)
N1—C1—C2	123.1 (3)	C48—C47—H47	120.1
C2—C1—H1	118.4	C47—C48—H48	120.7
C1—C2—H2	120.3	C49—C48—C47	118.6 (3)
C1—C2—C3	119.3 (3)	C49—C48—H48	120.7
C3—C2—H2	120.3	C48—C49—H49	120.8
C2—C3—H3	120.8	C48—C49—C50	118.4 (3)
C4—C3—C2	118.4 (3)	C50—C49—H49	120.8
C4—C3—H3	120.8	N10—C50—C49	123.9 (3)
C3—C4—H4	120.4	N10—C50—H50	118.0
C3—C4—C5	119.2 (3)	C49—C50—H50	118.0
C5—C4—H4	120.4	N11—C51—H51	118.5
N1—C5—C4	122.8 (3)	N11—C51—C52	123.0 (4)
N1—C5—H5	118.6	C52—C51—H51	118.5
C4—C5—H5	118.6	C51—C52—H52	120.2
N2—C6—H6	118.8	C51—C52—C53	119.5 (4)
N2—C6—C7	122.4 (3)	C53—C52—H52	120.2
C7—C6—H6	118.8	C52—C53—H53	121.1
C6—C7—H7	120.2	C54—C53—C52	117.8 (4)
C8—C7—C6	119.5 (3)	C54—C53—H53	121.1
C8—C7—H7	120.2	C53—C54—H54	120.6
C7—C8—H8	120.8	C53—C54—C55	118.7 (4)
C7—C8—C9	118.4 (3)	C55—C54—H54	120.6
C9—C8—H8	120.8	N11—C55—C54	124.4 (3)
C8—C9—H9	120.5	N11—C55—H55	117.8
C8—C9—C10	118.9 (3)	C54—C55—H55	117.8
			
Co1—N1—C1—C2	172.6 (3)	C1—N1—C5—C4	0.7 (5)
Co1—N1—C5—C4	−173.2 (2)	C1—C2—C3—C4	−0.2 (6)
Co1—N2—C6—C7	−178.3 (2)	C2—C3—C4—C5	−0.5 (5)
Co1—N2—C10—C9	175.8 (2)	C3—C4—C5—N1	0.3 (5)
Co1—N3—C11—C12	−178.1 (2)	C5—N1—C1—C2	−1.5 (5)
Co1—N3—C15—C14	177.7 (3)	C6—N2—C10—C9	−2.5 (5)
Co1—N4—C16—C17	176.7 (3)	C6—C7—C8—C9	−2.7 (5)
Co1—N4—C20—C19	−178.7 (3)	C7—C8—C9—C10	0.3 (5)
Co2—N5—C21—C22	176.7 (2)	C8—C9—C10—N2	2.4 (5)
Co2—N5—C25—C24	−175.2 (2)	C10—N2—C6—C7	0.0 (4)
Co2—N6—C26—C27	176.1 (4)	C11—N3—C15—C14	−0.8 (5)
Co2—N6—C30—C29	−176.9 (2)	C11—C12—C13—C14	0.1 (5)
Co2—N7—C31—C32	178.5 (2)	C12—C13—C14—C15	−0.5 (5)
Co2—N7—C35—C34	−179.5 (3)	C13—C14—C15—N3	0.9 (5)
Co2—N8—C36—C37	177.2 (3)	C15—N3—C11—C12	0.3 (4)
Co2—N8—C40—C39	−178.0 (3)	C16—N4—C20—C19	−1.7 (5)
Co3^ii^—S1—O2—Co1	154.7 (2)	C16—C17—C18—C19	−1.1 (6)
Co3—N9—C41—C42	−174.8 (3)	C17—C18—C19—C20	−0.6 (6)
Co3—N9—C45—C44	172.2 (3)	C18—C19—C20—N4	2.1 (6)
Co3—N10—C46—C47	175.5 (3)	C20—N4—C16—C17	−0.2 (5)
Co3—N10—C50—C49	−175.4 (3)	C21—N5—C25—C24	1.3 (4)
Co3—N11—C51—C52	175.2 (4)	C21—C22—C23—C24	1.4 (5)
Co3—N11—C55—C54	−172.6 (3)	C22—C23—C24—C25	−0.1 (5)
O1—S1—O2—Co1	96.6 (3)	C23—C24—C25—N5	−1.3 (5)
O1—S1—O4—Co3^ii^	1.42 (13)	C25—N5—C21—C22	0.0 (4)
O2—S1—O1—Co3^ii^	114.25 (11)	C26—N6—C30—C29	0.9 (5)
O2—S1—O4—Co3^ii^	−115.04 (11)	C26—C27—C28—C29	1.4 (8)
O3—S1—O1—Co3^ii^	−122.30 (12)	C27—C28—C29—C30	−2.1 (7)
O3—S1—O2—Co1	−27.8 (3)	C28—C29—C30—N6	1.0 (6)
O3—S1—O4—Co3^ii^	122.92 (13)	C30—N6—C26—C27	−1.7 (6)
O4—S1—O1—Co3^ii^	−1.38 (13)	C31—N7—C35—C34	0.6 (5)
O4—S1—O2—Co1	−150.3 (3)	C31—C32—C33—C34	−0.3 (5)
O5—S2—O6—Co2	−91.4 (6)	C32—C33—C34—C35	−0.6 (6)
O6—S2—O5—Co1	−155.9 (3)	C33—C34—C35—N7	0.5 (6)
O7—S2—O5—Co1	86.4 (4)	C35—N7—C31—C32	−1.6 (5)
O7—S2—O6—Co2	25.6 (6)	C36—N8—C40—C39	−0.9 (5)
O8—S2—O5—Co1	−36.4 (4)	C36—C37—C38—C39	−0.2 (7)
O8—S2—O6—Co2	148.4 (6)	C37—C38—C39—C40	−0.7 (6)
O9—S3—O10—Co3	129.1 (3)	C38—C39—C40—N8	1.3 (6)
O10—S3—O9—Co2	−150.5 (3)	C40—N8—C36—C37	0.0 (5)
O11—S3—O9—Co2	−31.4 (4)	C41—N9—C45—C44	−2.3 (5)
O11—S3—O10—Co3	10.0 (3)	C41—C42—C43—C44	−2.0 (6)
O12—S3—O9—Co2	91.7 (3)	C42—C43—C44—C45	−0.2 (6)
O12—S3—O10—Co3	−113.4 (3)	C43—C44—C45—N9	2.5 (6)
N1—C1—C2—C3	1.3 (6)	C45—N9—C41—C42	−0.1 (5)
N2—C6—C7—C8	2.6 (5)	C46—N10—C50—C49	0.2 (5)
N3—C11—C12—C13	0.0 (5)	C46—C47—C48—C49	0.8 (5)
N4—C16—C17—C18	1.6 (6)	C47—C48—C49—C50	−0.6 (5)
N5—C21—C22—C23	−1.4 (5)	C48—C49—C50—N10	0.1 (5)
N6—C26—C27—C28	0.6 (8)	C50—N10—C46—C47	0.0 (4)
N7—C31—C32—C33	1.5 (5)	C51—N11—C55—C54	2.3 (6)
N8—C36—C37—C38	0.6 (6)	C51—C52—C53—C54	3.0 (8)
N9—C41—C42—C43	2.3 (6)	C52—C53—C54—C55	−0.8 (7)
N10—C46—C47—C48	−0.5 (5)	C53—C54—C55—N11	−1.9 (7)
N11—C51—C52—C53	−2.8 (8)	C55—N11—C51—C52	0.1 (6)

Symmetry codes: (i) −*x*+1/2, −*y*+1, *z*−1/2; (ii) −*x*+1/2, −*y*+1, *z*+1/2.

### catena-Poly[[tetrakis(pyridine-κN)cobalt(II)]-µ-sulfato-κ2O:O'-[tetrakis(pyridine-κN)cobalt(II)]-µ-sulfato-κ3O,O':O''-[tris(pyridine-κN)cobalt(II)]-µ-sulfato-κ2O:O'] (2) Hydrogen-bond geometry (Å, º)

**Table:**

*D*—H···*A*	*D*—H	H···*A*	*D*···*A*	*D*—H···*A*
C1—H1···O1	0.95	2.56	3.421 (4)	150
C1—H1···O2	0.95	2.58	3.066 (4)	112
C4—H4···O11^iii^	0.95	2.47	3.158 (4)	129
C6—H6···O3	0.95	2.48	3.263 (4)	140
C15—H15···O5	0.95	2.47	2.967 (4)	113
C24—H24···O7^iv^	0.95	2.59	3.322 (4)	134
C26—H26···O11	0.95	2.40	3.343 (4)	171
C30—H30···O6	0.95	2.51	3.079 (4)	119
C30—H30···O7	0.95	2.50	3.161 (4)	126
C31—H31···O6	0.95	2.59	3.107 (4)	115
C35—H35···O9	0.95	2.36	2.936 (4)	119
C36—H36···O6	0.95	2.41	3.003 (4)	121
C40—H40···O12	0.95	2.43	3.352 (4)	163
C46—H46···O11	0.95	2.30	3.225 (4)	166
C50—H50···O4^i^	0.95	2.49	3.132 (4)	125
C51—H51···O10	0.95	2.46	3.019 (4)	117

Symmetry codes: (i) −*x*+1/2, −*y*+1, *z*−1/2; (iii) −*x*+1, *y*+1/2, −*z*+3/2; (iv) −*x*+1, *y*−1/2, −*z*+3/2.

## References

[bb1] Ali, H. M., Puvaneswary, S. & Ng, S. W. (2005). *Acta Cryst.* E**61**, m474–m475.

[bb2] Bruker (2016). *APEX3*, *SAINT* and *SADABS*. Bruker AXS Inc., Madison, Wisconsin, USA.

[bb3] Castiñeiras, A. & García-Santos, I. (2008). *Z. Anorg. Allg. Chem.* **634**, 2907–2916.

[bb4] Cotton, F. A., Daniels, L. M., Murillo, C. A. & Zúňiga, L. A. (1994). *Eur. J. Solid State Inorg. Chem.* **31**, 535–544.

[bb5] Cotton, F. A. & Reid, A. H. Jr (1984). *New J. Chem.* **8**, 203–206.

[bb6] Dolomanov, O. V., Bourhis, L. J., Gildea, R. J., Howard, J. A. K. & Puschmann, H. (2009). *J. Appl. Cryst.* **42**, 339–341.

[bb7] Haynes, J. S., Rettig, S. J., Sams, J. R., Thompson, R. C. & Trotter, J. (1986). *Can. J. Chem.* **64**, 429–441.

[bb8] Howe, J. L. (1898). *Science*, **8**, 945–947.

[bb9] Kožíšek, J., Hricov, A. & Langfelderová, H. (1989). *Acta Cryst.* C**45**, 885–887.

[bb10] Memon, A. A., Afzaal, M., Malik, M. A., Nguyen, C. Q., O’Brien, P. & Raftery, J. (2006). *Dalton Trans.* pp. 4499–4505. 10.1039/b606661e 16981025

[bb11] Parsons, S., Flack, H. D. & Wagner, T. (2013). *Acta Cryst.* B**69**, 249–259. 10.1107/S2052519213010014 PMC3661305 23719469

[bb12] Reitzenstein, F. (1894). *Justus Liebigs Ann. Chem.* **282**, 267–280.

[bb13] Reitzenstein, F. (1898). *Z. Anorg. Chem.* **18**, 253–304.

[bb14] Roy, M., Pham, D. N. K., Kreider-Mueller, A., Golen, J. A. & Manke, D. R. (2018). *Acta Cryst.* C**74**, 263–268. 10.1107/S2053229618001547 29504552

[bb15] Sheldrick, G. M. (2008). *Acta Cryst.* A**64**, 112–122. 10.1107/S0108767307043930 18156677

[bb16] Sheldrick, G. M. (2015). *Acta Cryst.* C**71**, 3–8.

[bb17] Shi, Y.-F., Li, F.-X., Geng, B., Liu, Y.-C. & Chen, Z.-F. (2009). *Acta Cryst.* E**65**, m1665. 10.1107/S1600536809049605 PMC2971987 21578675

[bb18] Westrip, S. P. (2010). *J. Appl. Cryst.* **43**, 920–925.

[bb19] Zhang, Y.-X. (2004). *Acta Cryst.* E**60**, m30–m31.

